# Supplementary material for: Trend of Ultraprocessed Product Intake Is Associated with the Double Burden of Malnutrition in Mexican Children and Adolescents
Source: Nutrients. 2022 Oct 17;14(20):4347. doi: 10.3390/nu14204347 (PMC9607204; doi:10.3390/nu14204347)
Supplement: Supplementary file 1 [file nutrients-14-04347-s001.zip › nutrients-1903915-supplementary.pdf]

**Definition of ultra-processed products:** Ultra-processed products are industrial formulations of ingredients that result from a series of industrial processes to enhance their sensory qualities. They are commonly nutritionally unbalanced and hyper-palatable.
Flavored prepared milk (chocolate or other flavor) or flavoring added to milk
Industrialized yogurt and dairy products
Sandwich, hamburger, pizza, and hot dog
Longaniza or chorizo, sausage, ham, or mortadella
Saltine crackers
Breakfast cereal, sweetened or light
Soda, regular or diet
Non-dairy coffee creamer
Industrialized flavored beverages or waters
Chocolate
Candies and chocolate
Spicy Mexican candy
Fried snack
Marshmallow
Jello, flan, cake, or pie
Ice cream
Microwaveable popcorn
Industrialized pastries and doughnuts
Sweetened cookies and cereal bars
Instant soups
Margarine and mayonnaise
Vegetable shortenings
Industrialized sauces and dressings
Industrialized wheat flour tortilla

Figure S1. Food and beverages of semi-quantitative food consumption frequency questionnaire classified as ultra-processed products.

Table S1. Characteristics of children by socioeconomic status in 2006 and 2016.

	Children					
	Year 2006			Year 2016		
Socioeconomic status [a]	Tertile 1	Tertile 2	Tertile 3	Tertile 1	Tertile 2	Tertile 3
Sample [b],	2953	2931	2190	1421	950	563
Total energy intake kcal/d, mean (95% CI)	1391, (1353, 1430)	1559, (1521, 1596)	1681, (1636, 1726)	1524, (1460, 1588)	1594, (1528, 1659)	1707, (1631, 1782)
Ultra-processed products						
kcal/d, mean (95% CI)	284, (263, 305)	464, (444, 483)	591, (564, 619)	379, (348, 409)	510, (470, 550)	617, (563, 670)
% of energy, mean (95% CI)	19.4, (18.2, 20.7)	29, (28, 30)	34.4, (33.3, 35.5)	23.9, (22.1, 25.7)	31.3, (29.4, 33.3)	35.6, (32.8, 38.4)
Age, mean (95% CI)	8.7, (8.6, 8.8)	8.8, (8.7, 8.9)	8.8, (8.6, 8.9)	7.9, (7.7, 8.1)	8.2, (8, 8.5)	7.9, (7.7, 8.2)
Sex, % (95% CI)						
Men	49.5, (46.6, 52.4)	49.7, (46.7, 52.7)	50, (46.6, 53.4)	47, (43.1, 51)	51, (45.7, 56.3)	56.6, (49.1, 63.7)
Women	50.5, (47.6, 53.4)	50.3, (47.3, 53.3)	50, (46.6, 53.4)	53, (49, 56.9)	49, (43.7, 54.3)	43.4, (36.3, 50.9)
Screen time, % (95%CI)						
less than 7 hr/week	83.5, (80.1, 86.4)	66.1, (60.5, 71.3)	47.2, (40.5, 54)	12.2, (8.5, 17.2)	6, (3, 11.5)	1.8, (0.5, 6.2)
7 to 13 hr/week	15.6, (12.7, 19)	31.9, (26.9, 37.3)	42.5, (35.9, 49.3)	25.7, (17.3, 36.2)	10.2, (6.3, 16)	5.2, (1.7, 14.8)
14 to 20 hr/week	0.9, (0.5, 1.7)	2, (1, 3.7)	9.2, (4.8, 16.8)	15.6, (10.4, 22.7)	17.1, (10.3, 26.9)	10.9, (5.9, 19.1)
21 or more hr/week	0, (0, 0)	0.1, (0, 0.3)	1.1, (0.3, 4.2)	46.5, (37, 56.3)	66.8, (57.5, 75)	82.2, (71.5, 89.4)
Head of household educational level [c]						
Low	80.9, (78.1, 83.4)	63, (59.6, 66.3)	42.3, (38, 46.8)	54.3, (49.1, 59.4)	36.6, (30.9, 42.8)	22, (16.1, 29.4)
Medium	18.8, (16.3, 21.6)	35.8, (32.6, 39.3)	45.8, (41.4, 50.3)	44.7, (39.6, 49.9)	61.4, (55.3, 67.2)	55.1, (46.7, 63.4)
High	0.3, (0.2, 0.6)	1.2, (0.8, 1.8)	11.9, (8.2, 16.9)	1, (0.5, 1.9)	1.9, (1, 3.5)	22.8, (14.4, 34.2)
Indigenism						
Indigenous	43.1, (38.2, 48.1)	16.3, (14, 18.9)	8.5, (7, 10.3)	9.7, (5.7, 16.1)	2, (1, 4)	0.5, (0.1, 1.9)
Nonindigenous	56.9, (51.9, 61.8)	83.7, (81.1, 86)	91.5, (89.7, 93)	90.3, (83.9, 94.3)	98, (96, 99)	99.5, (98.1, 99.9)
Area [d], % (95% CI)						
Urban	36.7, (32, 41.7)	62.8, (58.4, 67.1)	86.9, (84.2, 89.3)	52.7, (46.1, 59.3)	75.3, (69.1, 80.6)	86.7, (80.4, 91.1)
Rural	63.3, (58.3, 68)	37.2, (32.9, 41.6)	13.1, (10.7, 15.8)	47.3, (40.7, 53.9)	24.7, (19.4, 30.9)	13.3, (8.9, 19.6)
Region, % (95% CI)						
North	7.7, (4.9, 12.1)	14.2, (11.7, 17.3)	16.4, (13.4, 19.9)	16.2, (10.3, 24.6)	22.5, (16.8, 29.4)	23, (13.7, 36.1)
Central and Mexico City	30, (24.5, 36.1)	53, (47.6, 58.3)	68.8, (64, 73.2)	33.8, (28.5, 39.6)	51.7, (42.9, 60.3)	56.1, (44.9, 66.7)
South	62.3, (55.8, 68.4)	32.8, (28.2, 37.7)	14.8, (12.3, 17.8)	49.9, (43.2, 56.6)	25.9, (19.7, 33.2)	20.9, (13.8, 30.3)
Health conditions						
Only anemia [e]	10.4, (8.6, 12.4)	8.9, (7, 11.2)	8.8, (6.4, 12)	10.6, (8.2, 13.6)	9.1, (6.5, 12.6)	7.6, (5.2, 11)
Only overweight and obesity [f]	19.7, (17.5, 22.2)	30.9, (27.9, 34)	36.8, (32.9, 40.8)	19.1, (16, 22.6)	29.4, (23.5, 36.2)	41, (33.3, 49.2)
Double Burden of Malnutrition [g]	2.1, (1.5, 2.8)	3.3, (2.5, 4.3)	2.9, (2, 4.2)	4.5, (3, 6.9)	2.8, (1.5, 5.3)	2.2, (1.1, 4.1)
Health conditions						
Anemia [e]	12.4, (10.5, 14.6)	12.2, (10.1, 14.6)	11.7, (9.2, 14.9)	15.1, (12.4, 18.4)	11.9, (8.9, 15.8)	9.8, (6.9, 13.7)
Excess weight [f]	21.8, (19.4, 24.3)	34.2, (31.2, 37.4)	39.7, (35.7, 43.8)	23.6, (20.3, 27.2)	32.2, (25.9, 39.3)	43.2, (35.6, 51.2)
Double Burden of Malnutrition [g]	2.1, (1.5, 2.8)	3.3, (2.5, 4.3)	2.9, (2, 4.2)	4.5, (3, 6.9)	2.8, (1.5, 5.3)	2.2, (1.1, 4.1)

[a] Socioeconomic status was derived using the principal components method; the index score was divided into tertiles to represent a low, medium, and high socioeconomic status.

[b] Estimated population (thousand) by socioeconomic status; Tertile 1: Year 2006 N=3310.4, Year 2016 N=5370.5, Tertile 2: Year 2006 N=3056, Year 2016 N=5439.5 Tertile 3: Year 2006 N=2991, Year 2016 N=4967.5.

[c] Head of household educational level: Low—no formal education to elementary school; Medium—middle and high school education; and High—college and postgraduate courses.

[d] Area rural defined by 1. Rural localities with less than 2,500 habitants, and urban with 2,500 or more.

[e] Anemia: The Hb concentrations to diagnose anemia adjusted by altitude were <11.5g/dl for children 5 to 11 years old; <12.0 g/dl for adolescents 12 to 14 years old; <12.0 g/dl for females ≥ 15 years old, and <13.0 g/dl for males ≥ 15 years old.

[f] Excess weight: Overweight or Obesity. Children and adolescents with *z* scores above +1 were classified as overweight and obese with z scores above +2 standard deviations.

[g] Individuals with anemia and overweight or obesity.

Table S2. Characteristics of adolescents by socioeconomic status in 2006 and 2016.

	Adolescents					
	Year 2006			Year 2016		
Socioeconomic status [a]	Tertile 1	Tertile 2	Tertile 3	Tertile 1	Tertile 2	Tertile 3
Sample [b], n	2034	2409	2039	916	720	481
Total energy intake kcal/d, mean (95% CI)	1684, (1635, 1732)	1779, (1735, 1824)	1846, (1802, 1890)	1976, (1885, 2067)	2011, (1921, 2100)	2080, (1965, 2195)
Ultra-processed products						
kcal/d, mean (95% CI)	263, (244, 282)	428, (407, 449)	541, (519, 564)	491, (434, 549)	600, (552, 648)	675, (611, 739)
% of energy, mean (95% CI)	15.8, (14.6, 16.9)	23.8, (22.8, 24.9)	29, (28.1, 30)	23.7, (21.7, 25.6)	29.1, (27.4, 30.9)	32, (30, 33.9)
Age, mean (95% CI)	15, (14.8, 15.1)	15.1, (15, 15.3)	15.3, (15.2, 15.4)	15.2, (15, 15.4)	15.4, (15.2, 15.7)	15.4, (15.2, 15.7)
Sex, % (95% CI)						
Men	49.6, (46.4, 52.7)	48.4, (45.3, 51.6)	50.5, (46.6, 54.4)	48.4, (42.5, 54.4)	49.3, (43.5, 55.3)	51.6, (45.5, 57.7)
Women	50.4, (47.3, 53.6)	51.6, (48.4, 54.7)	49.5, (45.6, 53.4)	51.6, (45.6, 57.5)	50.7, (44.7, 56.5)	48.4, (42.3, 54.5)
Screen time, % (95%CI)						
less than 7 hr/week	77.7, (74.8, 80.4)	65.6, (62.7, 68.4)	48.8, (45.2, 52.4)	10, (7.2, 13.8)	4.6, (2.9, 7.2)	1.6, (0.8, 2.9)
7 to 13 hr/week	20.3, (17.7, 23.1)	29.9, (27.3, 32.7)	38.9, (35.7, 42.3)	19.3, (15, 24.4)	11.8, (8.6, 15.9)	4.2, (2.3, 7.4)
14 to 20 hr/week	1.8, (1.2, 2.7)	3.9, (2.9, 5.1)	9.7, (7.6, 12.2)	14.2, (10.1, 19.5)	13, (9.7, 17.2)	6.8, (4.3, 10.5)
21 or more hr/week	0.2, (0, 0.6)	0.6, (0.2, 2)	2.6, (1.2, 5.5)	56.5, (50.1, 62.8)	70.7, (65.7, 75.2)	87.5, (83.1, 90.9)
Head of household educational level [c]						
Low	87.7, (85.2, 89.7)	70, (66.7, 73.1)	46, (42.6, 49.5)	64.4, (58.2, 70.2)	49.3, (43.5, 55.1)	24.6, (19.5, 30.6)
Medium	12.1, (10, 14.5)	28.4, (25.4, 31.6)	43.9, (40.3, 47.5)	35.3, (29.5, 41.5)	47, (41.1, 53)	57.8, (51.4, 64)
High	0.2, (0.1, 0.6)	1.6, (0.9, 2.7)	10.1, (7.8, 13.1)	0.3, (0.1, 0.9)	3.7, (2.2, 6.1)	17.5, (11.6, 25.7)
Indigenism						
Indigenous	42.4, (37, 47.9)	18, (15.3, 20.9)	10.4, (8.6, 12.7)	9.9, (6.4, 15.1)	4, (1.8, 8.5)	0.4, (0.1, 1.2)
Nonindigenous	57.6, (52.1, 63)	82, (79.1, 84.7)	89.6, (87.3, 91.4)	90.1, (84.9, 93.6)	96, (91.5, 98.2)	99.6, (98.8, 99.9)
Area [d], % (95% CI)						
Urban	34.7, (29.7, 40)	57.8, (53.5, 62)	83, (79.8, 85.8)	51.7, (45, 58.3)	75.1, (70.4, 79.3)	87.7, (82.3, 91.6)
Rural	65.3, (60, 70.3)	42.2, (38, 46.5)	17, (14.2, 20.2)	48.3, (41.7, 55)	24.9, (20.7, 29.6)	12.3, (8.4, 17.7)
Region, % (95% CI)						
North	7, (4.7, 10.1)	14, (11.5, 17)	19, (16, 22.5)	10.2, (6.9, 14.8)	20, (14.9, 26.2)	20.8, (15.2, 27.7)
Central and Mexico City	27, (21.5, 33.4)	51.1, (45.8, 56.3)	63.8, (59.2, 68.2)	38.7, (32.2, 45.6)	51.8, (45.6, 58)	56.7, (48.4, 64.6)
South	66, (59.5, 72)	34.9, (30.2, 39.9)	17.1, (14.3, 20.3)	51.1, (44.3, 57.8)	28.2, (23, 34.1)	22.6, (15.5, 31.6)
Health conditions						
Only anemia [e]	7.2, (5.9, 8.8)	4.8, (3.8, 6)	5.2, (3.9, 6.8)	9, (6.4, 12.6)	9.9, (6.7, 14.4)	4.2, (2.7, 6.6)
Only overweight and obesity [f]	20.7, (18.2, 23.4)	29.3, (26.6, 32.2)	32.6, (29.6, 35.7)	26.7, (22.2, 31.8)	29.9, (25.2, 35.1)	42.3, (36.7, 48.2)
Double Burden of Malnutrition [g]	2.4, (1.6, 3.6)	3.7, (2.5, 5.2)	2.3, (1.4, 3.7)	4.2, (2.4, 7.3)	2.7, (1.6, 4.4)	1.9, (1, 3.5)
Health conditions						
Anemia [e]	9.6, (8, 11.3)	8.4, (6.9, 10.4)	7.4, (5.8, 9.5)	13.3, (9.9, 17.6)	12.6, (9.2, 17)	6.1, (4.2, 8.8)
Excess weight [f]	23.1, (20.3, 26)	33, (30.1, 36)	34.8, (31.8, 38)	31, (26, 36.4)	32.6, (27.8, 37.8)	44.2, (38.5, 50.1)
Double Burden of Malnutrition [g]	2.4, (1.6, 3.6)	3.7, (2.5, 5.2)	2.3, (1.4, 3.7)	4.2, (2.4, 7.3)	2.7, (1.6, 4.4)	1.9, (1, 3.5)

[a] Socioeconomic status was derived using the principal components method; the index score was divided into tertiles to represent a low, medium, and high socioeconomic status.
[b] Estimated population (thousand) by socioeconomic status; Tertile 1: Year 2006 N=2973.5, Year 2016 N=5044.3, Tertile 2: Year 2006 N=3097.3, Year 2016 N=5194.8 Tertile 3: Year 2006 N=3056.1, Year 2016 N=5210.6.
[c] Head of household educational level: Low—no formal education to elementary school; Medium—middle and high school education; and High—college and postgraduate courses.

[d] Area rural defined by 1. Rural localities with less than 2,500 habitants, and urban with 2,500 or more.

[e] Anemia: The Hb concentrations to diagnose anemia adjusted by altitude were <11.5g/dl for children 5 to 11 years old; <12.0 g/dl for adolescents 12 to 14 years old; <12.0 g/dl for females ≥ 15 years old, and <13.0 g/dl for males ≥ 15 years old.
[f] Excess weight: Overweight or Obesity. Children and adolescents with *z* scores above +1 were classified as overweight and obese with *z* scores above +2 standard deviations.

[g] Individuals with anemia and overweight or obesity.
